# Draft Genome Sequence of *Shewanella* sp. Strain BF02_Schw, Isolated from Blood Falls, a Feature Where Subglacial Brine Discharges to the Surface of Taylor Glacier, Antarctica

**DOI:** 10.1128/MRA.01141-20

**Published:** 2021-06-03

**Authors:** Bruce W. Boles, Robert W. Murdoch, Ingemar Ohlsson, Jill A. Mikucki

**Affiliations:** a Department of Microbiology, University of Tennessee, Knoxville, Tennessee, USA; b Center for Environmental Biotechnology, University of Tennessee, Knoxville, Tennessee, USA; University of Delaware

## Abstract

We report the sequencing, assembly, and draft genome of *Shewanella* sp. strain BF02_Schw. The assembly contains 5,304,243 bp, with a GC content of 41.43%.

## ANNOUNCEMENT

**FIG 1:**
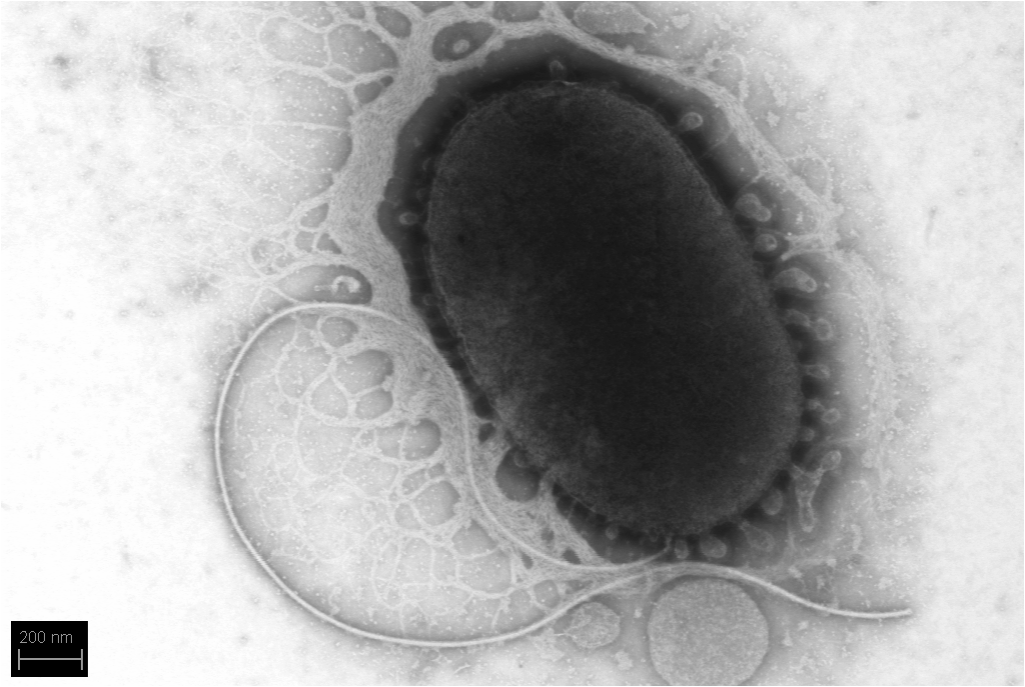
Image of *Shewanella* sp. strain BF02_Schw captured using the Zeiss Auriga dual-beam focused ion beam/scanning electron microscope at the University of Tennessee Joint Institute for Advanced Materials. Cells were rinsed in phosphate-buffered saline and pipetted onto a 200-mesh copper grid with a thin carbon film treated in a weak plasma. The sample was coated in a drop of 1% uranyl acetate, dried, and imaged at 30 kV in scanning transmission electron microscope mode.

Materials collected in November 2002 from the surface discharge of subglacial brine (1), a feature known as Blood Falls, in the McMurdo Dry Valleys of Antarctica (2, 3), were enriched at 4°C in minimal marine salts medium (1) and then streaked for isolation onto marine agar 2166 (BD 212185). Strain BF02_Schw is rod shaped with a monotrichous flagellum (Fig. 1).

Biomass for nucleic acid extraction was grown aerobically in marine broth 2166 (Difco) at 10°C for 5 days. Genomic DNA for long-read sequencing was extracted using the PowerSoil DNA isolation kit (Mo Bio Laboratories, Carlsbad, CA) according to the manufacturer’s protocols. Raw genomic DNA extract was prepared according to Oxford Nanopore Technologies (ONT; Oxford, England) protocols using the 1D rapid sequencing kit (SQK-RAD002). The prepared extract was applied to a new, primed FLO-MIN106 R9.4 SpotON flow cell connected to an ONT MinION sequencer.

Genomic DNA for Illumina sequencing was extracted using cetyltrimethylammonium bromide (CTAB) extraction (4). The sample library was prepared at MR DNA (Shallowater, TX) with the Nextera DNA preparation kit (Illumina, San Diego, CA) and was sequenced using paired ends for 300 cycles with a HiSeq system (Illumina).

Default parameters were used for all software unless otherwise specified. Sequence data were acquired from the MinION system with MinKNOW (v1.2.8) software. ONT sequences were base called using Albacore (v2.3.1; ONT), and sequences designated as passing were used in the hybrid assembly.

ONT sequencing yielded 63,821 reads and a total of 136,727,549 bases, with an average read length of 2,142.36 bp. Illumina sequencing yielded 5,833,395 reads, with an average length of 133.7 bp; forward and reverse sequences produced 780,169,484 bp and 779,728,149 bp, respectively. Prior to assembly, raw Illumina sequences were subsampled to ∼50× coverage using the seqtk toolkit (v1.3) (https://github.com/lh3/seqtk). Trimmomatic (v0.38) was used to perform quality trimming and to remove residual read-through adapter sequences (5).

Hybrid assembly of ONT and Illumina quality-controlled sequences using Unicycler (v0.4.8) (6) produced 29 contigs, with a total length of 5,304,243 bp and a GC content of 41.43%. An average coverage of 72.3× was determined using minimap2 (v2.17) (7). The assembly *N*_50_ and *L*_50_ values were 1,017,345 bp and 2 contigs, respectively. Comparison of the 16S rRNA gene from strain BF02_Schw using the Basic Local Alignment Search Tool (BLAST) (8) indicated >99% sequence identity with the *Shewanella* genus (1).

### Data availability.

This whole-genome shotgun project has been deposited in DDBJ/ENA/GenBank under the accession number JABKAW000000000. The version described in this paper is version JABKAW000000000.2. Raw sequence data are available in the NCBI Sequence Read Archive (SRA) under the accession numbers SRR12187017 and SRR12187018, as part of BioProject number PRJNA632589.
